# Neuroinflammation as a target for treatment of stroke using mesenchymal stem cells and extracellular vesicles

**DOI:** 10.1186/s12974-019-1571-8

**Published:** 2019-09-12

**Authors:** Sylwia Dabrowska, Anna Andrzejewska, Barbara Lukomska, Miroslaw Janowski

**Affiliations:** 1 NeuroRepair Department, Mossakowski Medical Research Centre, PAS, 5 Pawinskiego Street, 02-106 Warsaw, Poland; 2 Department of Diagnostic Radiology and Nuclear Medicine, University of Maryland, Baltimore, HSF III, 620 W. Baltimore street, Baltimore, MD 21201 USA

**Keywords:** Stroke, Ischemia, Neuro-inflammation, Mesenchymal stem cells, Extracellular vesicles

## Abstract

Ischemic stroke is the third cause of death in the developed countries and the main reason of severe disability. Brain ischemia leads to the production of damage-associated molecular patterns (DAMPs) by neurons and glial cells which results in astrocyte and microglia activation, pro-inflammatory cytokines and chemokines production, blood-brain barrier (BBB) disruption, infiltration of leukocytes from the peripheral blood into the infarcted area, and further exacerbation of tissue damage. However, some immune cells such as microglia or monocytes are capable to change their phenotype to anti-inflammatory, produce anti-inflammatory cytokines, and protect injured nervous tissue. In this situation, therapies, which will modulate the immune response after brain ischemia, such as transplantation of mesenchymal stem cells (MSCs) are catching interest. Many experimental studies of ischemic stroke revealed that MSCs are able to modulate immune response and act neuroprotective, through stimulation of neurogenesis, oligodendrogenesis, astrogenesis, and angiogenesis. MSCs may also have an ability to replace injured cells, but the release of paracrine factors directly into the environment or via extracellular vesicles (EVs) seems to play the most pronounced role. EVs are membrane structures containing proteins, lipids, and nucleic acids, and they express similar properties as the cells from which they are derived. However, EVs have lower immunogenicity, do not express the risk of vessel blockage, and have the capacity to cross the blood-brain barrier. Experimental studies of ischemic stroke showed that EVs have immunomodulatory and neuroprotective properties; therefore, they can stimulate neurogenesis and angiogenesis. Up to now, 20 clinical trials with MSC transplantation into patients after stroke were performed, from which two concerned on only hemorrhagic stroke and 13 studied only on ischemic stroke. There is no clinical trial with EV injection into patients after brain ischemia so far, but the case with miR-124-enriched EVs administration is planned and probably there will be more clinical studies with EV transplantation in the near future.

## Introduction

Stem cell-based regenerative medicine is quickly catching attention, and there is an accumulation of data that positive effects of stem cell therapy frequently depend on their immunomodulatory properties. Stroke induces an extensive neuro-inflammatory response, which seems to be responsible for the propagation of brain damage. Therefore, there is a link between stem cells and stroke, which centers on inflammation, and it has a high potential to be exploited in both basic research and clinical setting.

### Brain ischemia

Brain ischemia is one of the most important pathologies of the central nervous system (CNS). Ischemic stroke accounts for 87% of all stroke cases and it is the third most frequent cause of death people over 60 years old in developed countries and the leading cause of severe disability. It is estimated that every year, 15 million people in the world are affected by stroke, 5 million of which die and another 5 million suffer from long-term disability [1]. World statistics show that stroke affects women more often than men and is the second cause of death of females after 60 years old and represent 60% of all deaths caused by stroke [2]. According to the available data, 3–7% of all health care funds in developed countries are allocated for the treatment of people with stroke [3]. In ischemic stroke, serious damage of the nervous tissue occurs as a result of blocking the blood supply to the brain with subsequent insufficiency in the delivery of oxygen and nutrients [1]. The main factors increasing the incidence of ischemic stroke are hypertension, coronary heart disease, diabetes, smoking, hypercholesterolemia, transient ischemic attack, and atrial fibrillation [4].

During brain ischemia, the damage of the nervous tissue is observed in two areas—“ischemic core” in which the blood flow is lower than 10 mL/100 g/min and where the death of most cells occurs, and “ischemic penumbra” in which the blood flow is 10–20 mL/100 g/min, no neuronal death is observed but changes in tissue structure are visible. Oxygen and glucose deprivation in the area of the ischemic core leads to the reduction of neuronal adenosine triphosphate (ATP) production, which causes a decrease in the ionic gradient along the cell membrane and an increase in the Na^+^ ion level and Ca^2+^ in the cytoplasm. Glutamate accumulation and *N*-methyl-*D*-aspartate (NMDA) and α-amino-3-hydroxy-5-methyl-4-isoxazolepropionic acid (AMPA) receptor activation lead to a further influx of Ca^2+^ ions to the cells [1]. These processes result in the damage of cytoplasmic cell membrane, destruction of cell structures, activation of “inflammatory” cascade, and apoptosis and necrosis of cells [5]. In the ischemic penumbra, an increase in the level of glutamate derived from the ischemic core induces an increase in Ca^2+^ ions and Ca^2+^-dependent enzymes which activates the production of apoptosis mediators such as nitric oxide, free radicals, or arachidonic acid [1]. These processes can initiate programmed cell death or necrosis depending on the magnitude of damage and the metabolic state of the cells.

Currently, to treat patients after ischemic stroke, reperfusion therapy with thrombolytic drugs such as intravenous tissue plasminogen activator (tPA) or mechanical thrombectomy (MT) is used. Unfortunately, these therapies have many limitations, such as a narrow therapeutic window, which is up to 4.5 h from the onset of ischemic stroke in the case of tPA and up to 6–8 h in the case of MT, with only a limited number of cases benefiting from the extended time window till 24 h [6]. In addition, the tissue plasminogen activator is not effective for patients, who have high-level artery occlusion, when the thrombus is large or the stroke is extensive [7]. The rehabilitation of patients after ischemic stroke is frequently insufficiently effective and in many cases does not restore lost functions. Hence, there is a continuous search for new therapeutic strategies aimed at protecting neurons in the area of penumbra, preventing further cell damage during tissue reperfusion in the acute phase of the stroke, as well as the replacement of dead cells [5]. The current research is focused not only on the elimination of pathophysiological processes occurring in neural cells after ischemic stroke, but also on the modulation of local inflammatory response to ischemia.

### Inflammatory contribution to ischemic brain damage

The organism reacts to brain ischemia through induction of local and systemic inflammation in the absence of infectious pathogens, which is called “sterile inflammation.”

#### Local cellular reactions induced by brain ischemia

**Fig. 1:**
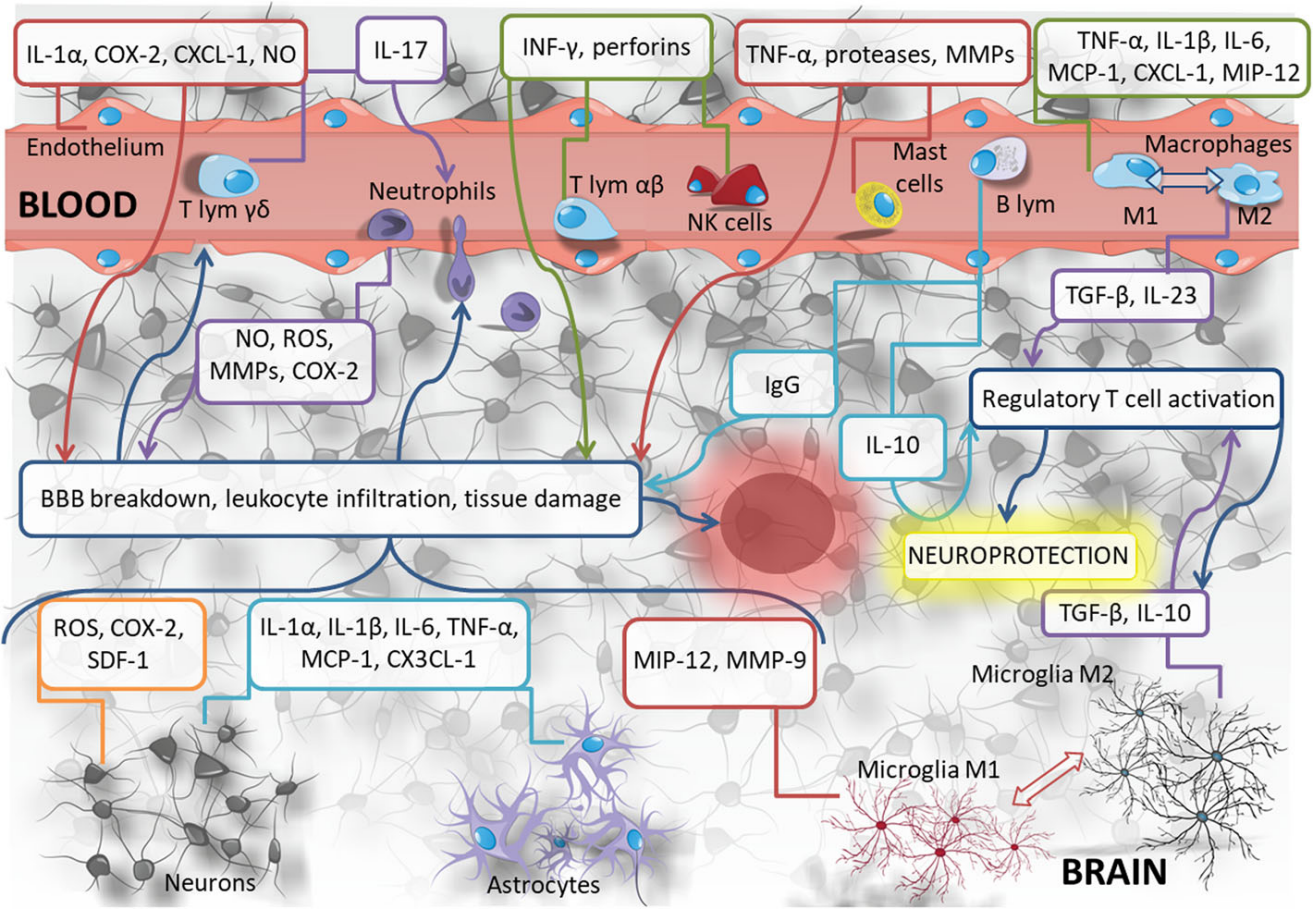
Inflammatory reactions accompanying brain ischemia

Shortly after the onset of ischemic stroke, the insulted neurons and glial cells produce DAMPs, which lead to astrocyte activation up to 28 days after the ischemic episode (Fig. 1). Activated astrocytes are capable of rapid proliferation and they change their shape and functions [8]. Upon activation, they secrete pro-inflammatory cytokines, chemokines, and metalloproteinases [9]. Factors released from astrocytes, including interleukin-1β (IL-1β) and matrix metalloproteinases (MMPs), contribute to the damage of the blood-brain barrier and increased infiltration of leukocytes from the blood to the nervous tissue [10, 11]. The inflow of these cells leads to further progression of damage in the subacute phase of stroke and so-called secondary damage. Astrocytes can also play a positive role in the response to ischemia. As a result of polarization, they change their phenotype and are capable to uptake extracellular glutamate as well as secrete neurotrophic factors, thus to protect the damaged brain tissue [11].

Microglial cells, similarly as astrocytes, are also the first line of the innate immune response in the CNS and are activated a few minutes after ischemic stroke onset (Fig. 1). The peak of activation is usually visible 48–72 h after the onset of ischemia, and this activation persists up to several weeks after tissue damage [12]. Activated microglia change their shape to amoeboid and acquire phagocytic capacity [8]. Quiescent microglial cells residing in the brain may become antigen-presenting cells (APC) to T cells as well as they can enhance T cell response to the major histocompatibility complex (MHC) class II antigens by increasing the expression of additional molecules, including CD40, CD80, and CD86 [13]. Analogically to astrocytes, microglia may both activate and inhibit the immune response in the nervous tissue after brain ischemia. Microglial cells with the pro-inflammatory M1 phenotype secrete pro-inflammatory mediators such as interleukin-1 (IL-1), tumor necrosis factor α (TNF-α), and MMP-9, which are involved in the leakage of the blood-brain barrier. Polarization of microglial cell phenotype from pro-inflammatory M1-type to anti-inflammatory M2-type results in the release of anti-inflammatory cytokines, including transforming growth factor β (TGF-β) and interleukin-10 (IL-10), and activates T regulatory cells (CD4^+^ CD25^+^), which modulate immune reactions and reduce inflammatory response [14].

Activation of astrocytes and microglia cells and the factors released by them cause further disruption of the blood-brain barrier and increased migration of leukocytes from the peripheral blood [15] as shown in Fig. 1. An increased inflow of monocytes to the damaged tissue is observed within the first 24 h after cerebral ischemia. There are two subpopulations of monocytes: CD14^+^CD16^−^ cells with pro-inflammatory properties and CD14^+^CD16^+^ monocytes with anti-inflammatory properties [16]. Increased number of monocytes is observed up to 7 days after stroke [17]. Some studies revealed that the proportion of pro-inflammatory monocytes decreases over time, while the percentage of anti-inflammatory monocytes increases. This is accompanied by the decrease in pro-inflammatory mediators such as chemokine from the group CC (β) ligand 2 (CCL2), and the increase in anti-inflammatory factors, including TGF-β1 [16]. M2-type macrophages are becoming predominant in the damaged brain tissue over time. Interestingly, they originate from pro-inflammatory monocytes (CD14^+^CD16^−^) migrating from the bloodstream in the early stages after stroke [18]. However, there are some contrary studies showing the polarization of anti-inflammatory M2-type macrophages towards pro-inflammatory M1-type macrophages in subacute and chronic phases of cerebral ischemia [19]. Nevertheless, the transformation of M1-type macrophages (CD16^+^CD32^+^CD86^+^) towards M2-type macrophages (CD206^+^Arginase-1^+^) is essential for the induction of tissue regenerative processes. M2-type macrophages, known for its immune-regulatory properties in existing in vitro tests [20], play an important role in endogenous repair and neuroprotection processes of neural tissue damaged by ischemia [18]. Another cell type involved in innate immune responses induced by ischemia in the brain is neutrophils, which appear in the damaged brain tissue immediately after stroke [21]. They accumulate in the vicinity of the ischemic area and release pro-inflammatory cytokines, free oxygen radicals, and proteolytic enzymes, which cause additional destruction of the nervous tissue, especially in the area of the penumbra surrounding the lesion core [11, 22]. The number of neutrophils present in the brain after ischemia corresponds directly to the size of ischemic damage [8]. Increased neutrophil influx from the vascular bed is the result of the production of MMP-3 and MMP-9 by them as well as BBB disruption [23]. The latest research by Neumann et al. shows that the interaction of neutrophils with microglial cells suppresses the neuroprotective effect of the latter and increases the area of the lesion after stroke [24]. Neutrophil phagocytosis by microglial cells in the nervous tissue may be an important factor in reducing ischemia-induced damage [24, 25]. Fast-acting innate immune response also induces mechanisms of adaptive immune response. Peripheral blood-originated lymphocytes play a pivotal role in adaptive immune response and both T and B lymphocytes infiltrate the brain after ischemia.

There are both αβ T cells and γδ T cells observed in the neural tissue damaged by stroke [26]. The cytotoxicity of the perforins released by CD8^+^ T cells and the effect of IL-21 produced by CD4^+^ T lymphocytes are important direct mechanisms of damage associated with the action of αβ T lymphocytes [27, 28]. In turn, regulatory T cells (Tregs) show protective properties, especially in the later phase after stroke. It seems that the positive effect is associated with the release of IL-10 and TGF-β [29] as well as with inhibitory effect of Tregs on the production of MMP-9 by neutrophils [30]. However, other authors suggest that regulatory T cells may have an adverse effect, causing microvascular dysfunction in the damaged tissue [31].

Recent reports suggest that regulatory B cells to a greater extent than regulatory T lymphocytes suppress the inflow of pro-inflammatory cells to the site of injury. μMT (−/−) mice with B cell deficits showed a larger area of the lesion after brain ischemia and a much longer time to improve neurological functions than wild-type mice [32]. B lymphocytes in the acute phase of stroke are protective against damage of nervous tissue by release of IL-10, which inhibits the production of pro-inflammatory cytokines by T lymphocytes [32]. However, the suppression of immune responses after stroke by B cells is questioned by other authors. The studies performed by Elvington et al. on a murine model of ischemic stroke indicate a pathological role of immunoglobulins accumulating in the nervous tissue, including autoantibodies against phospholipids, which induce neuronal death and increase the area of damages as late as 4–7 weeks after ischemia [33]. It has also been shown that the accumulation of antibodies correlates with impaired function of the hippocampus, which results in short-term memory disturbances within a few weeks after the onset of ischemic stroke [34]. However, in the case of intravenous administration of immunoglobulins, blocking of complement protein activation and a clear neuroprotective effect in a model of brain ischemia are observed [35].

A number of papers emphasize the significant contribution of γδ T cells in the immune response after stroke. The effector mechanisms of γδ T cells are associated with the production of interleukin-17 (IL-17) [23]. The synergistic stimulation of astrocytes by IL-17 and TNF-α, released after ischemia by activated microglial cells and macrophages, causes neutrophil infiltration as a response to an increased amount of chemokines in the damaged tissue, including the CXC group CXCL-1 chemokine. Blocking IL-17 with antibodies or inhibiting the activation of CXCL-1/CXCR2 (CXC group receptor 2) decreases the inflammatory response after stroke and reduces the lesion area [36].

Ischemic stroke causes also natural killer (NK) cell infiltration into the brain. It seems that NK cells are involved in the early phase of the immune response to tissue damage. Neuronal death has been shown to be associated with the effects of interferon-γ (IFN γ) and perforins released by NK cells 3 h after reperfusion [36–38]. Dendritic cells (DCs) are involved in the immune response after brain ischemia as well. It has been shown that in addition to their classical role in the antigen-dependent response, DCs are able to modulate local tissue reactions, regardless of migration to lymphoid organs or antigen presentation [39, 40]. A significant number of positive CD11c cells appear 24 h after stroke induction and it peaks at 3 days. Dendritic cells present in the damaged hemisphere are characterized by CD172a^+^/IRF4^+^ phenotype. The interleukin-23 (IL-23) produced by them induces the release of IL-17 from γδ T cells and, as a consequence, neutrophil infiltration into the damaged tissue [41].

Mast cells present in the meninges and cerebral blood vessels are also involved in the inflammatory reactions after ischemic stroke. Mastocytes secrete cytoplasmic granules containing a vasoactive substance, histamine; anticoagulant, heparin; TNF-α; and proteases, e.g., tryptase, chymase, MMP-2, and MMP-9, contributing to the damage of the blood-brain barrier, cerebral edema, and neutrophil infiltration to the damaged nervous tissue [42, 43]. Mast cells may phagocytose, serve as antigen-presenting cells, and modulate the mechanisms of secondary immune response [44].

#### Mediators of inflammatory reactions

Mediators of inflammatory processes such as cytokines and chemokines are also observed in the ischemic area. These proteins are secreted by activated cells present in the brain such as neurons and glial cells and cells migrating from the peripheral blood after an occurrence of ischemia. Interleukin-1 (IL-1) is the main pro-inflammatory cytokine that appears several hours after ischemic brain damage [45]. IL-1α is mainly produced by not only microglial cells [45], but also astrocytes, endothelial cells, and neurons [10]. IL-1 stimulates astrocytes to secrete cytokines and chemokines and to increase the production of MMP-9, which causes the blood-brain barrier disruption [46]. In addition, it affects endothelial cells, by increasing the expression of adhesion molecules, mainly intercellular adhesion molecule 1 (ICAM-1) and vascular cell adhesion molecules 1 (VCAM-1), which initiates neutrophil penetration into the damaged tissue [47].

In the initial phase after stroke, interleukin-6 (IL-6) appears in the damaged brain tissue [48] and its elevated level is observed up to 12 months after ischemia [49]. In the nervous tissue, IL-6 is produced by microglia cells, macrophages derived from peripheral blood monocytes, astrocytes and neurons [50]. IL-6 was originally thought to be a pro-inflammatory cytokine, but based on recent research, it can play a dual role in response to ischemia. It contributes to the brain injury through T and B lymphocyte stimulation and activation of acute-phase proteins [48]. Other studies have shown that IL-6 can be neuroprotective [51, 52]. Biological activity of IL-6 enhances the activity of IL-1, which may also inhibit its pro-inflammatory effect by the synthesis of its receptor antagonist (IL-1Ra) [53].

Important cytokines with pro-inflammatory effects, which appear in the brain as a result of ischemia, are interleukin 17A (IL-17A) and interleukin 23 (IL-23). IL-17A is secreted by γδ T cells as a result of their activation through a cascade of cytokines released by αβ T lymphocytes and microglial cells following brain damage, and it initiates neutrophil infiltration [36, 54]. Previous studies have shown that blocking IL-17A with specific antibodies is neuroprotective in a murine model of ischemic stroke [36]. IL-23 is produced by M2 macrophages and dendritic cells. Its elevated level in the brain is already observed 24 h after the onset of ischemia. The main action of IL-23 is the activation of γδ T lymphocytes [23]. The inhibition of the IL-23/IL-17 cascade limits the stroke size after stroke [41].

Additionally, to the growth of IL-1, IL-6, IL-17, and IL-23 observed a few hours after ischemia, there is an increase of TNF-α. It seems that TNF-α is responsible for initiating the inflammatory process after brain injury [55]. In the initial period after stroke, TNF-α is produced by neurons and in a later phase by microglial cells, astrocytes, lymphocytes, and macrophages derived from the peripheral blood [37]. Elevated TNF-α level in the blood and cerebrospinal fluid of patients is found 24 h after the onset of ischemic stroke and lasts for 1–2 weeks [55]. TNF-α causes apoptosis of neurons, enlargement of the damaged area, and the increase of neurological deficits [56]. TNF-α also induces the migration of leukocytes from the vascular bed to the vicinity of the lesion [37]. Experimental studies have shown that specific anti-TNF-α antibodies administered to the ventricular system before induction of ischemic episode have neuroprotective effects limiting the area of injury [57].

Another pro-inflammatory cytokine involved in reactions after ischemia of the brain is interferon gamma produced by leukocytes, mainly by αβ T cells and NK cells [58]. Experimental studies have shown an elevated level of INF-γ 24–72 h after the ischemic stroke onset. The main action of INF-γ is the activation of chemokine interferon-γ inducible protein 10 (IP-10/CXCL10) secretion [59]. Systemic administration of anti-INF-γ antibodies to animals after stroke reduces T cell infiltration in injured tissue and limits the damage [60]. The interleukin-4 (IL-4) observed in the brain after ischemia performs regulatory functions. It is mainly produced by not only αβ T cells, but also NK cells, mast cells [61], and damaged neurons [62]. The main role of IL-4 is to control the differentiation of Th2 lymphocytes and B lymphocytes. CNS studies have shown that IL-4 stimulates the conversion of microglial/macrophage cells from the M1 pro-inflammatory phenotype into anti-inflammatory cells with the M2 phenotype, thus protecting against nervous tissue damage [62].

Among anti-inflammatory cytokines, there is an increase in interleukin-10 during cerebral ischemia. Interestingly, this is accompanied by the reduction of IL-10 in the peripheral blood of patients in the acute phase of the stroke within the first 12–24 h [63, 64]. IL-10 is released by microglia cells with M2 phenotype, activated astrocytes, and regulatory T cells. This cytokine inhibits the synthesis of pro-inflammatory factors, promotes the survival of neurons and glial cells, acts protectively, and stimulates neuro-regeneration [14, 65]. Experimental studies have shown that the administration of IL-10 to rats with focal cerebral hypoxia results in the reduction of the tissue damage [66]. Data on the neuroprotective effects of IL-10 has also been confirmed in clinical trials [67].

Transforming growth factor β (TGF-β) is another cytokine playing a beneficial role in the immune response after stroke. Elevated level of TGF-β is observed in patients’ blood at the first day after ischemic stroke [68]. In the experimental models of brain ischemia, the increase in TGF-β was evident within the first hours of the onset of an ischemic episode. In damaged nervous tissue, TGF-β is produced by M2 phenotype microglia cells and regulatory T lymphocytes [69]. The neuroprotective effect of TGF-β is caused by an activation of many mechanisms to protect neurons against death, including reduction of damage associated with excitotoxicity of glutamate; secretion of chemokines, including monocyte chemoattractant protein (MCP-1) and macrophage inflammatory protein1α (MIP-1α); and inhibition of apoptosis in the damaged nervous tissue [11, 70].

The increase in cytokine activity in the nervous tissue after brain ischemia is accompanied by an increase in the level of chemokines—heparin-binding proteins of 8–14 kDa mass, acting through surface receptors belonging to the G-protein-coupled receptor superfamily (GPCRs). Due to its construction, four groups of chemokines are distinguished: CXC (α), CC (β), CX3C (δ), and CX3C (γ). In the immune response after stroke, chemokines show both pro-and anti-inflammatory activities, and their role is to recruit leukocytes to the site of injury and stimulate immunologically active cells [71].

One of the chemokines, which is present after cerebral ischemia, is the chemokine group CXC ligand 12 (CXCL12), also called stromal cell-derived factor (SDF-1), which is mainly produced by damaged neurons. Studies have shown that CXCL12 stimulates pro-inflammatory response in the acute phase of ischemic stroke by binding to the CXCR4 receptor. Blocking CXCL12 or its receptor results in reduced leukocyte infiltration to the lesion site, decreased production of pro-inflammatory cytokines, and reduced blood-brain barrier damage [72, 73]. In the later phases of stroke, CXCL12 plays a beneficial role by activating processes of neurogenesis and angiogenesis [74] and protection of newly formed neurons [75].

The chemokine released during the acute phase of ischemic stroke is chemokine group CXC ligand 1 (CXCL1), also known as GRO or KC, secreted mainly by macrophages and endothelial cells. A significant increase in its level was found in cerebrospinal fluid 24 h after ischemia [76] and in the blood serum of patients [77]. The main function of CXCL1 is to stimulate neutrophil and immunologically competent cell infiltration to the site of injury, which results in an increase of inflammatory reactions and nervous cell death in the brain ischemia area [78].

Another chemokine following ischemic stroke is CC (β) ligand 2 (CCL2) chemokine, also called a monocyte chemotactic protein-1 (MCP-1), produced by microglia cells, astrocytes, neurons, and inflowing leukocytes. Significant increase of MCP-1 level is observed a few hours after brain ischemia and lasts for several days after ischemia [79]. In the acute phase of ischemic stroke, MCP-1 has a pro-inflammatory effect. Studies have shown that blocking of chemokine or its receptor CCR2 reduced monocyte infiltration to the damaged tissue, decreased production of pro-inflammatory cytokines, and the reduced blood-brain barrier permeability and lesion area [80, 81]. The positive effect of MCP-1 in the regeneration of damaged tissue has been demonstrated long time after the onset of ischemia. Liu et al. proved that MCP-1 participates in neurogenesis processes by recruiting neuroblasts from the sub-chamber zone to the lesion site [82] and maintaining the integrity of neurovascular unit [16].

Macrophage inflammatory proteins (MIPs), MIP-1α (CCL3) and MIP-3α (CCL20), are also involved in inflammatory response after ischemic stroke. MIP-1α is secreted mainly by microglial cells and monocytes, and its highest level is observed between 8 and 72 h after ischemia. The most important activities of MIP-1α include recruitment of monocytes into damaged nervous tissue and activation of astrocytes and microglial cells [83]. MIP-3α is released mainly by astrocytes in the presence of IL-1β and TNFα and it stimulates IL-1β and nitric oxide synthase production. Experiments by Terao et al. showed that blocking of MIP-3α secretion results in a reduction of the damaged area in the rat model of brain ischemia [84].

Another chemokine produced in large quantities by neurons, astrocytes, microglia cells and incoming macrophages, T cells, and NK cells is the chemokine group CX3C ligand 1 (CX3CL1) called fractalkine or neurotactin. Experimental studies on a murine model of brain ischemia have shown that blocking CX3CL1 or its receptor CX3CR1 results in reduction of negative effects of tissue ischemia, including excitotoxicity reactions, production of reactive oxygen species (ROS), pro-inflammatory cytokines release, blood-brain barrier damage, leukocyte infiltration, and apoptosis of cells in the injured area [85, 86]. Rosito et al. noticed that fractalkine stimulated the secretion of the chemokine group CXC ligand 16 (CXCL16) by glial cells which induced protection of damaged nervous tissue and reduction of excitotoxicity induced by the presence of glutamate [87].

Many enzymes are involved in the immune response after ischemic stroke. The most important are matrix metalloproteinases, including MMP-2 and MMP-9, whose level and activity increases in a short time after ischemia. MMPs are involved in the depletion of the blood-brain barrier as well as the destruction of myelin sheaths [88]. It seems that in turn in chronic phase of stroke metalloproteinases play a beneficial role by activating the production of vascular endothelial growth factor (VEGF), which is important in the process of neovascularization and the conditioning of the environment [89]. Ischemic stroke leads to increased production of cyclooxygenase (COX-2) induced by neurons, neutrophils, and endothelial cells. COX-2 causes an increase in inflammatory reactions through participation in the formation of toxic prostanoids and peroxides. Its blockage results in a reduction of the blood-brain barrier damage and limitation of leukocyte migration from the blood to the brain [90]. Ischemia also induces the expression of nitric oxide synthase (NOS), which causes the release of large amounts of nitric oxide (NO) by neutrophils, microglia cells, macrophages, and endothelial cells. In the initial stage after stroke, NO plays a beneficial role by inducing vasodilation, while in the later stages, it intensifies nervous tissue damage [91]. Recent studies indicate that the synthesis of nitric oxide via environmental conditioning may act neuroprotective in the area of ischemia [92].

### Inflammatory reactions after hemorrhagic stroke

Primary brain damage after hemorrhagic stroke is mainly caused by the sudden increase of intracerebral pressure by rapid extravasation of blood. Secondary injury after hemorrhagic stroke is related to the induction of inflammatory reactions due to the negative impact of the gradual decay of blood components on the surrounding brain tissue [93]. The systemic effectors of the immune response such as inflammatory cells, cytokines, chemokines, and proteases leak to the brain and contribute to the progression of the injury [93, 94]. Thrombin activates cytotoxic, excitotoxic, and immune reactions which cause edema formation, blood-brain barrier damage, and leukocytes infiltration [95, 96]. Hemoglobin released from red blood cells initiates the generation of free radicals which lead to oxidative damage and induce inflammatory reactions [97]. Hemin causes the increase of iron concentration, decrease of glutathione, and release of free radicals leading to further tissue damage [95].

Few minutes after the onset of hemorrhagic stroke, microglial cells are activated. Microglia cells are the first type of inflammatory cells involved in immune response after hemorrhagic stroke [98]. They can act neuroprotectively by clearing the hematoma and debris of damaged cells through phagocytosis. However, microglia cells may also spur the immune reactions after hemorrhagic stroke by releasing of pro-inflammatory mediators such as TNF-α, IL-1β, NOS, or MMP-9 which leads to infiltration of leukocytes and further progression of the tissue damage [98, 99].

Neutrophils are the first type of leukocytes, which infiltrate to the injured brain after hemorrhagic stroke. They appear in the early stage after injury and achieve the highest number 3 days after the hemorrhagic damage [100]. Recent studies performed on a rat model of collagenase-induced hemorrhagic stroke revealed that neutrophil depletion reduced MMP-9 expression, blood vessels disruption, blood-brain barrier leakage, axon damage, and astrocyte and microglial/macrophage activation [101].

Another type of leukocytes contributing to the immune response after hemorrhagic stroke is CD8+ T cells and CD4+ T cells. The studies on animal models revealed that the number of CD8+ lymphocytes increased 24 h after the injury and achieve the highest level 2–7 days after the onset of hemorrhagic stroke [102]. Another experimental work showed that the elevated number of CD4+ T lymphocytes appeared 4 days after induction of hemorrhagic stroke [103]. The decrease of both types of T cells caused by fingolimod led to the improvement of neurological functions and edema reduction 24 and 48 h after hemorrhagic stroke as well as a decrease of pro-inflammatory mediators: interferon-γ, IL-17, and intracellular adhesion molecule-1 [104].

Astrocytes are another type of cells playing an important role in inflammatory reactions after hemorrhagic stroke. The experimental studies showed that the number of reactive astrocytes in the peri-hematomal region is elevated from 1 to 7 days after the onset of hemorrhagic stroke [105]. Activated astrocytes induce pro-inflammatory reactions and participate in edema mainly by induction of MMP-9 [106]. Moreover, Mestriner et al. revealed the similar plasticity of activated astrocytes in perilesional sensorimotor cortex and striatum after ischemic and hemorrhagic stroke [107].

### Experimental therapy of brain ischemia using MSCs and EVs

Experimental therapies aimed at reducing immunological reactions after ischemic stroke using immunologically active cell inhibitors or mediators of inflammation have not been successful so far. In this situation, new therapeutic strategies using immunomodulation mechanisms are sought, one of which is transplantation of mesenchymal stem cells or extracellular vesicles derived from them.

**Fig. 2:**
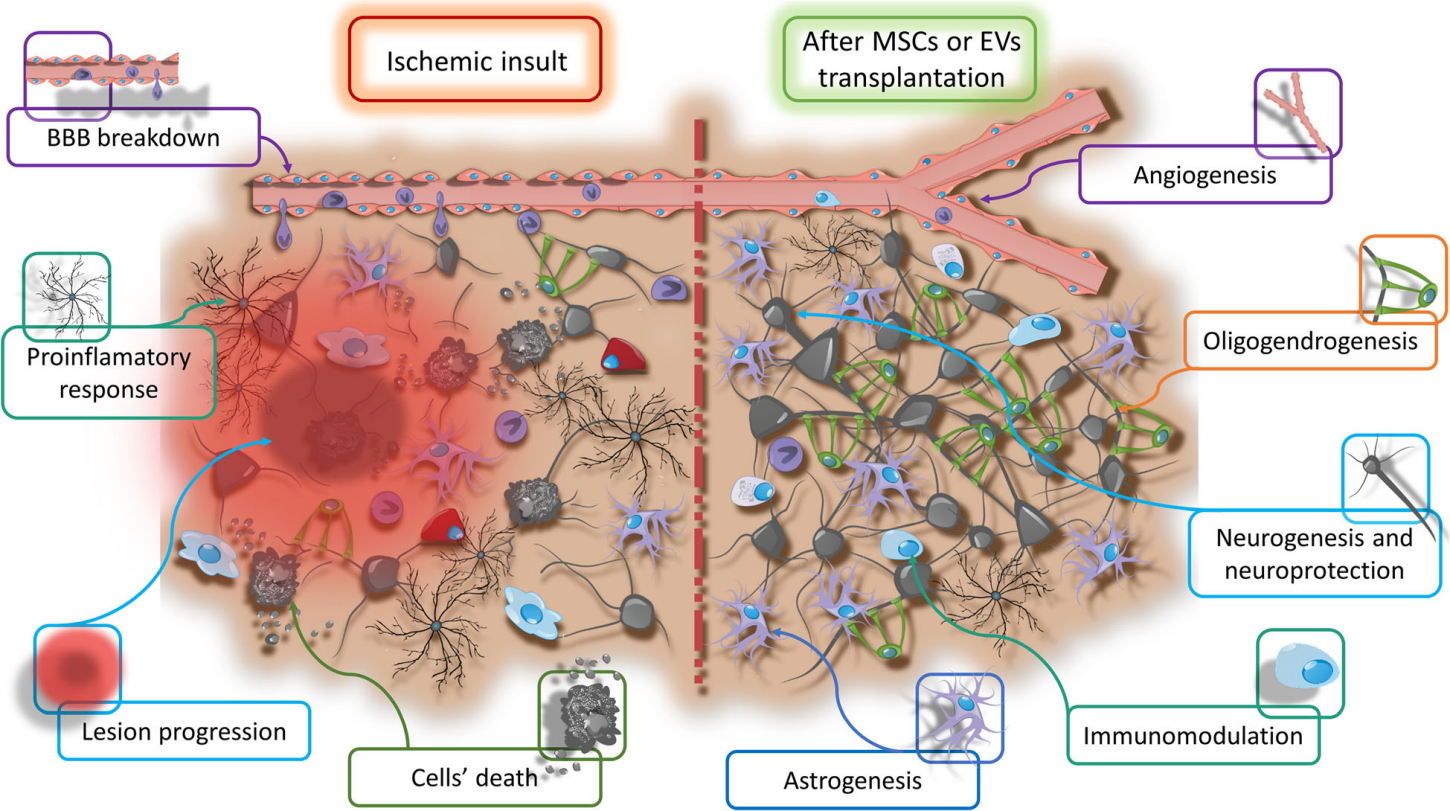
Processes regulated after MSCs or EVs transplantation in an experimental therapy of brain ischemia

There are various mechanisms of action involved in potential therapeutic activity of mesenchymal stem cells including neuroprotection, immunomodulation, and stimulation of a new synapse formation as well as activation of neurogenesis, astrogenesis, oligodendrogenesis, and angiogenesis (Fig. 2). Current research suggests that this therapeutic effect is mainly related to the impact of the MSC secretome on endogenous stem cells and host microenvironment and to a lesser extent the direct differentiation of MSCs into neural cells [5, 108].

#### MSCs in the experimental therapy of brain ischemia using in vitro models

The results of experimental studies in brain ischemia models, in vitro, showed that even a short-term presence of MSCs results in a positive effect on neurons at the site of injury and reduction of unfavorable inflammatory processes. In vitro ischemic studies of the brain were performed using hypoxia- and glutamate-induced excitotoxicity models and oxygen and glucose deprivation (OGD). The work of Huang et al. showed that co-culture of MSCs with neural N17 cells previously subjected to oxygen and glucose deprivation restores the prolonged proliferation of N17 cells as a result of OGD, reduces their apoptosis, and decreases TNF-α level which proves the protective and anti-inflammatory effect of MSCs [109]. Experiments performed using neurons or neuroblastoma cells have shown that MSCs reduce the death of these cells caused by ischemia in both experiments with co-cultures of cells and using supernatants from the MSCs culture, indicating on the neuroprotective properties of mesenchymal stem cells [110, 111].

In studies conducted by other authors using mouse spinal cord explants, primary retinal ganglion or brain neuron motifs, the presence of MSCs induces neurite growth in the basal ganglia (DRG) and survival of retinal ganglion cells (RGCs), cortical and dopaminergic neurons [5, 112, 113]. Positive effects of MSCs have been demonstrated by Hung et al. who added a supernatant from the MSC culture to the primary culture of human endothelial cells isolated from the aorta. It turned out that the addition of MSCs supernatant to endothelial cells damaged by ischemia increases their survival, reduces apoptosis, and stimulates the process of angiogenesis [114]. The increased level of VEGF and MCP-1 derived from MSCs in conditioned media was probably responsible for the anti-apoptotic and pro-angiogenic effects.

#### MSCs in the experimental therapy of brain ischemia using in vivo animal models

Results of mesenchymal stem cells obtained in experiments in vitro were verified in in vivo experiments using animal models. A series of structural studies were performed to evaluate the therapeutic properties of MSCs and functional and behavioral tests, using numerous experimental animal models, many types of MSCs, and various delivery routes. A common method of cell transplantation into the brain after ischemic damage is intracerebral surgery. As one of the first, Zhao et al. showed that bone marrow mesenchymal stem cells (BM-MSCs) transplanted into the rat’s cerebral cortex migrated to the area of damage were able to survive in the host’s brain, differentiated into mature neurons, and assisted in the restoration of lost functions, which was confirmed by behavioral tests [115].

In other studies, it was observed that after transplantation, MSCs can differentiate into neural cells, although more often into astroglial cells than into neurons. However, they did not show the ability to migration or further differentiation, so it is difficult to suppose that new neural cells derived from MSCs were responsible for the functional improvement of animals. The positive effect of transplantation was probably related to the paracrine activity of MSCs. This effect was demonstrated in the experiments of Leong and Liao groups, who observed that MSCs transplanted intracerebrally in the rat model of stroke induce neuronal activity, reduce cell death, and stimulate angiogenesis [116, 117]. In addition, MSCs transplanted intracerebrally can modulate the immune response associated with cerebral ischemia. The factors they produce, including TGF-β, inhibit the secretion of MCP-1 and limit the infiltration of CD68^+^ cells to the damaged tissue [118, 119].

The intracerebral injection of MSCs is a relatively invasive procedure, which is why the systemic routes of application are increasingly used for transplantations. The most commonly used route of MSC systemic administration in the model of brain ischemic stroke is intravenous transplantation. Intravenous transplantation of MSCs in a rat model of brain ischemia increased also a proliferation of neural progenitors in the subventricular zone (SVZ) as well as the number of oligodendrocytes, neurofilaments, and synapses which is accompanied by a simultaneous increase in the level of synaptophysin and VEGF [120, 121]. Studies involving MSCs derived from embryonic cells have shown that their intravenous administration resulted in their migration to the area of cerebral ischemia. Therefore, after colonization, they expressed neural markers and endothelial cell antigens and were neuroprotective [122]. According to Lee et al., MSCs administered intravenously in a rat model of ischemic stroke to stimulate the effects of SDF-1/CXCR4, which is important in the area of damage they infest [123]. In some experiments, after intravenous administration of MSCs, there were not any cells in damaged tissues or their number was very small, although transplanted MSCs were responsible for the improvement of impaired functions in stroke.

Intra-arterial route of cell delivery to the brain in the animal model of brain ischemia has been increasingly used [124]. Compared to intravenous administration, it turned out that intra-arterially transplanted MSCs were present in the cerebral cortex and peripheries of the lesion, expressed astrocyte and neuronal markers, and induced faster improvement in neurological function in animals [125–127]. Other studies on animal models have shown that despite the small number of MSCs visible in the area of cerebral ischemia after their transplantation, increased axonal growth and re-myelination [128], stimulation of angiogenesis with a simultaneous decrease in microglial cell activity, and decrease in MMP-9 were observed [125]. Beneficial effects of intra-arterially transplanted MSCs in the rat model of brain ischemia, including an increased number of axons, were observed up to 1 year after transplantation [127]. Genetic analysis revealed the presence of exogenous MSCs of males in the female brain which co-expressed astrocytes, neurons, and endothelial cell markers. Transplanted cells were also found in other recipient organs. Attempts at intra-arterial transplantation of MSCs were also carried out in large animals. Mesenchymal stem cells derived from human umbilical cord blood transplanted in the dog model of brain ischemia were observed in the area of injury, and they expressed markers typical for neurons and astrocytes. After MSC injection, a smaller area of damage and faster improvement of motor functions were noted in comparison with animals that did not receive a transplant [129]. Immunomodulatory properties of intra-arterially transplanted MSCs were demonstrated in the rat brain injured by stroke, where a statistically significant decrease in IL-2 mRNA and IL-6 mRNA levels was observed [130]. At the same time, transplant recipients showed a decrease of the lesion area and reduction in neurological symptoms caused by an episode of cerebral ischemia. Acosta et al. observed that BM-MSCs administered intravenously in the rat model of chronic ischemic stroke migrated to the spleen. A decrease in the number of cells expressing MHC class II antigens and reduction in TNF-α level indicates modulation of the general immune response, which may result in a 30% reduction of the damaged area [131]. The advances in precision of intra-arterial administration through advanced imaging using real-time magnetic resonance imaging (MRI) further attract this route of cell delivery [132].

Therapeutic effects of MSCs in the animal model of brain ischemia were also observed after intranasal administration. Wei et al. have shown that transplantation of hypoxic BM-MSCs with increased expression of migration-related proteins such as CXCR4, MMP-2, and MMP-9 results in more efficient colonization of ischemic damaged tissue by transplanted MSCs as well as increased cell survival in the lesion periphery [133]. Intranasal injection can also be effective and a minimally invasive way of cell administration in newborns. Experiments performed by van Velthoven et al. revealed that after intranasal transplantation of MSCs in suckling rats after ischemia shows a smaller area of damage, increased proliferation of neural progenitors in the injured hemisphere, and quicker restoration of motor functions [134]. In addition, after intranasal administration of BM-MSCs to rats in the model of brain ischemia, there is an increase in angiogenesis, faster recovery of the neurovascular unit, improvement of blood flow in the cerebral cortex, and return of sensory-motor and olfactory functions [135].

#### MSC-derived extracellular vesicles (EVs) in the experimental therapy of brain ischemia

It has been recently shown that the positive effects of MSCs are at least partially related to the production of EVs [136]. EVs contain membrane receptors and proteins, lipids, and nucleic acids including various forms of RNA and can be used to treat various diseases instead of cells, showing a similar effect. The biggest advantages of EV application are low immunogenicity; lower risk of vessel blockage and microvascular thrombosis; potentially better ability to cross the blood-brain barrier after systemic transplantation; the ease of creating a large-scale production of genetically modified EVs; a higher surface to volume ratio and the associated stronger transfer of active particles to the target tissues; and relatively easy genetic modification of the miRNAs contained in EVs. The therapeutic effect of EVs in brain ischemia can be related to the modulation of a number of processes, including induction of neurogenesis, activation of angiogenesis, inhibition of apoptosis, modulation of the immune response, and reprogramming of cells [137] (Fig. 2**)**.

In vitro studies using cortical neuron cultures have shown that EVs isolated from MSCs stimulate the growth of nervous cells [138]. EVs present at supernatants derived from MSC culture act neuroprotective through increasing of neuronal survival as well as stimulation of neural cell regeneration in the model of glutamate-induced excitotoxicity [139]. Lin and co-workers proved that EVs isolated from MSCs protect rat PC12 cells from damage caused by glutamate through activation of the PI3K/Akt pathway [140]. Experiments conducted by another group have shown that EVs derived from MSCs isolated from fat cause an increase in CδII protein kinase expression in the immortalized mouse hippocampal cell line and induce neuronal proliferation [141].

Due to the small size and the ability to cross the blood-brain barrier, EVs are often transplanted systemically. EVs from MSCs transplanted into rats 24 h after induction of brain ischemia activate endogenous neurogenesis, increase the number of axons, and improve vital functions of animals [142]. Moreover, after the administration of EVs isolated from MSCs, long-term neuroprotection and modulation of the peripheral immune response have been observed [143]. Similarly, the transplantation of EVs released from MSCs in a model of brain ischemia in sheep fetuses was neuroprotective. In these animals, the reduction of the lesion area and general improvement of functions impaired after ischemia were observed, which gives hope for the treatment of hypoxic-ischemic brain damage in newborns [144]. Chen et al. revealed that EVs isolated from adipose-derived mesenchymal stem cells (AD-MSCs) have similar properties to those derived from bone marrow. Transplantation of EVs from AD-MSCs into rats 3 h after brain ischemia resulted in the decrease of the damaged area, modulation of the immune response, and improvement of neurological function [145]. Otero-Ortega et al. showed that the EVs implanted intravenously in the rats with subcortical stroke model improve signal transduction, axonal growth, and white matter restoration as well as reduce motor deficits caused by ischemia [146]. Until now, there are no published reports of intra-arterial delivery of EVs in a model of ischemic stroke, but there are the recent reports of EV labeling for detection in MRI [147, 148].

EVs have also an impact on angiogenesis in the brain after ischemia. Xin et al. demonstrated that EVs injected intravenously into the rat model of stroke increase the expression of von Willebrand factor (vWF) in the area of injury [142]. The results of other authors indicate the activation of long-lasting angiogenesis in the mouse brain after intravenous transplantation of EVs from MSCs [143]. In turn, Chen et al. observed an increased expression of markers related to angiogenesis, including CD31, vWF, and VEGF and the growth in the number of small vessels in the injured area after intravenous transplantation of EVs isolated from AD-MSCs, which significantly improved the blood flow in the rat brain after ischemic stroke [145]. Positive effects of EV transplantation in cerebral ischemia models were also observed using EVs derived from non-MSCs cell types. Hayon et al. showed that intravenously transplanted EVs isolated from platelets increased proliferation of endogenous neural cells, induced angiogenesis, and improved general motor and cognitive functions in rats after ischemic stroke [149]. EVs from platelets contain growth factors that stimulate the proliferation of neural progenitor cells and the restoration of the neurovascular unit, which can be used in the therapy of brain ischemia [149, 150]. Furthermore, it has been shown that the secretome of apoptotic mononuclear cells isolated from the peripheral blood of healthy donors transplanted into rats after ischemic stroke have neuroprotective effects, reduce brain damage, and restore neurological functions impaired after ischemia [151].

One of the main mechanisms responsible for the therapeutic properties of EVs injected in the brain ischemia is the transfer of miRNAs between cells. In vitro experiments have shown that EVs from MSCs stimulate axonal growth through the process in which miRNA is involved [138]. In vivo studies confirmed that EVs modulate responses after ischemic stroke by transferring miRNA. EVs from MSCs contain miRNA which affect post-transcriptional gene regulation and expression of proteins in target cells, contributing to faster improvement of neurological functions in animals subjected to ischemic stroke. Various types of miRNAs are involved in these processes, including miR-133b [152]. Experiments have shown that EVs from MSCs stimulate endogenous brain cells to produce miRNAs, affecting brain plasticity after ischemia [137]. In addition, Huang and colleagues have shown that the increase of miR-124-3p in EVs derived from microglial cells activates the polarity of these cells towards the anti-inflammatory phenotype, inhibits pro-inflammatory reactions, and induces the reconstruction of damaged neurons [153]. At present, there are also attempts to use genetically modified EVs. In vitro studies with the OGD model have proven that EVs from astrocytes treated with EVs isolated from MSCs with miR-133b overexpression significantly increase neurite growth in primary cortical neuronal cultures as compared to EVs derived from control cells [154]. Xin et al. also showed that EVs isolated from MSCs enriched with miRNA-17-92 significantly improve neurological functions, enhance neurogenesis and oligodendrogenesis, and increase dendritic plasticity in rats after brain ischemia compared to EVs lacking additional miRNA [155]. The same group revealed that EVs derived from MSCs with overexpression of mi-R-133b are more potent than EVs derived from control cells in enhancing brain plasticity and to return lost functions in the rat model of stroke [154]. Additional EV modifications, e.g., intra-nasally transplanted EVs derived from embryonic stem cells encapsulated in curcumin reduce activation of astrocytes, increase the expression of a nuclear-specific protein for mature neurons (NeuN) and endothelial tight junctions in the mice model of ischemic stroke [156].

### MSCs in human clinical stroke studies

**Fig. 3:**
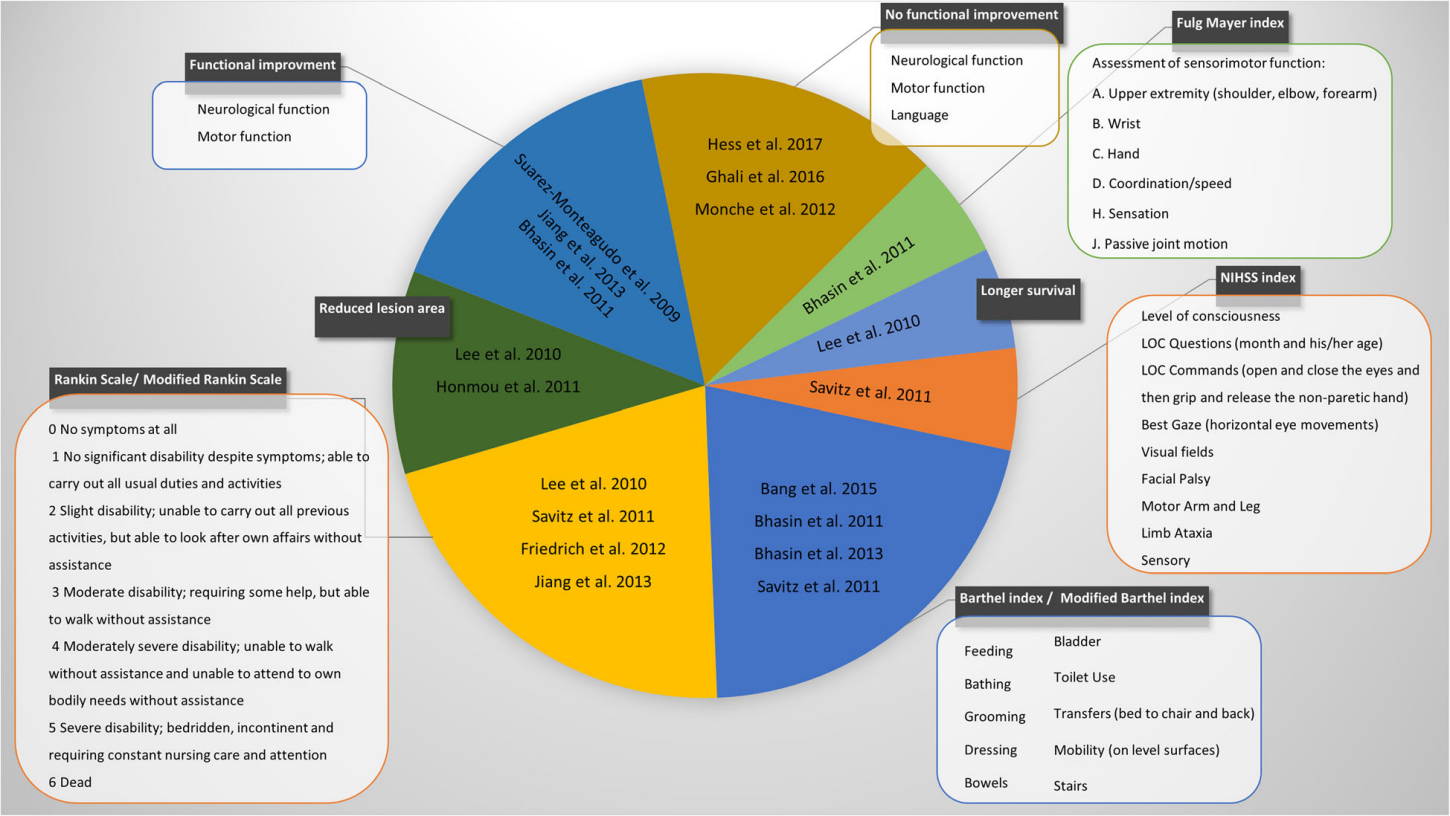
Positive changes in outcome measure in clinical trials of MSC transplantation into patients with brain ischemia

The main principles of MSC application in clinical trials with patients after stroke were established at a meeting of scientists, representatives of companies, and members of the National Institutes of Health named “Stem Cell Therapies as an Emerging Paradigm for Stroke (STEPS)” [157]. It has been considered that prior to clinical translation, ethical, technical, and medical problems should be solved. From an ethical and safety point of view, the use of MSCs is more justified than an application of embryonic stem cells as they are derived from adult tissues so are ethically neutral and possess a lower risk of neoplastic transformation. Technical problems are related to the determination of an appropriate time needed for isolation as well as proliferation and characterization of MSCs. It is also necessary to determine the route of MSC transplantation. Intracerebral administration brings beneficial effects; however, it is more invasive than intravascular cell transplantation. Another problem is choosing the right time between stroke and MSCs administration, as well as the adequate number of cells and the frequency of applications. It is also necessary to confirm the lack of negative influence of MSCs on other patients’ illnesses and medicines, as well as to demonstrate the rationale for using genetically modified cells [108]. Despite these difficulties, the beneficial results obtained in experimental animal studies justify their transfer to the clinical phase and the transplantation into patients with brain ischemia. So far, 20 clinical trials have been carried out using MSCs in stroke patients, of which only 2 were hemorrhagic stroke and 13 were attempted with ischemic stroke [158] (Fig. 3). In most studies, MSCs derived from the bone marrow were administered systemically.

One of the first phase I/II clinical trials was conducted by Bang et al. who transplanted BM-MSCs intravenously, twice within 9 weeks after ischemic stroke. The study included five patients undergoing MSC transplantation and 25 control patients, and the total dose of given cells was 1 × 10^8^ BM-MSCs. In the group of people after BM-MSC transplantation, there were not any deaths caused by cell transplantation, recurrence or post-transplant abnormalities, while a significant improvement in Barthel’s Index in comparison with control patients was noticed during the first year of observation [159]. Lee et al. performed a 5-year clinical study with two intravenous administrations of autologous BM-MSCs in 85 patients after ischemic stroke. Interestingly, there was an observed much longer survival of patients, which received MSCs (72% of patients), as compared to the control group (34% of patients). The improvement of the clinical condition in patients who received MSCs was accompanied by an increase of the SDF-1 level in the blood and a smaller degree of damage in the sub-granular zone of the lateral ventricles [160].

Bhasin et al. conducted clinical two trials using intravenous transplantation of BM-MSCs into people with chronic ischemic stroke and hemorrhagic stroke. The first study involved 12 people, of whom six received 5–6x10^7^cells and was followed up till 24 weeks. There were no deaths or significant side effects in the group of treated patients, while a slight increase in the Fugl-Meyer index and the modified Barthel index was recorded as well as an increase in the activation of Brodman BA4 and BA6 fields demonstrating enhanced neuroplasticity in patients who received BM-MSCs [161]. The second clinical trial was carried out according to the same protocol but the number of people was increased to 40 subjects. In this group of patients, no side effects or deaths due to transplanted cells were also observed. From all of the tested parameters, only a statistically significant increase in the modified Barthel index was found [162]. Honmou and colleagues conducted a clinical trial in 12 people with white and gray matter injuries resulting from past ischemic stroke. All patients were administered intravenously 0.6–1.6 × 10^8^ autologous BM-MSCs from 36 to 133 days after the onset of cerebral ischemia. No adverse effects of transplanted cells were observed and MSC transplantation caused a reduction in the lesion area by at least 20% within 1 week after BM-MSCs injection [163]. Savitz et al. administered intravenously 8.5 × 10^7^ bone marrow mononuclear cells (BM-MNCs) to 11 patients between 7 and 30 days after the stroke. Cell infusion was carried out within 4 h after bone marrow aspiration. There were no side effects, while statistically significant improvement in the following factors was observed: National Institute of Health Stroke Scale (NIHSS), Barthel index, and the modified Rankin Scale [157].

In recent years, Hess et al. conducted a randomized, double-blinded, phase II clinical trial using placebo control in patients with ischemic stroke within 24–48 h of an ischemic episode. The study was conducted in 129 people, of whom 67 patients received intravenous infusion of multipotent adult bone marrow progenitor cells, commercially available from Lonza, and compared to 62 subjects who received a placebo. During the 90 days of the study, no side effects were observed; however, no significant functional improvement was found in the group of patients who received the cells in relation to patients who received placebo [164]. Suárez-Monteagudo et al. performed a clinical trial with stereotactic administration of BM-MNCs into five people after ischemic and hemorrhagic stroke, which occurred in the thalamus, striatum, and primary motoric cortex. Patients were transplanted to the brain from 1.73 × 10^7^ up to 5.5 × 10^7^ autologous BM-MNCs. During 1 year of follow-up, all patients with MNC transplantation showed a slight functional improvement and no side effects [165].

Currently, the number of clinical trials with intra-arterial administration is quickly increasing. Barbosa da Fonseca et al. performed a clinical trial in six people with 59–82-day-old ischemic stroke. Patients received 1.25–5 × 10^8^ autologous bone marrow mononuclear cells intra-arterially, of which 2 × 10^7^ cells were labeled with 99 mTc. BM-MNCs were present in the brain for up to 24 h after administration, and there were also observed in the liver, lungs, spleen, kidneys, and bladder. No adverse effects were found after 120 days of analysis [166]. Similarly, the safety of the cell application was confirmed by Battistella et al. The authors administered intra-arterially 1–5 × 10^8^ autologous BM-MNCs to six people. During the 180 days of observation conducted in patients who received a transplant of cells, they did not observe any side effects [167].

Ghali et al. conducted a clinical trial in 39 patients in the subacute phase of ischemic stroke, 21 of whom received intra-arterially autologous mononuclear cells derived from the bone marrow and 18 people served as a control group. BM-MNC graft did not induce any significant adverse effects, but in these patients, no significant improvement of motor skills, reduction of speech disorders, or reduction of the brain injury area was observed compared to the control group [168]. Friedrich et al. performed intra-arterial BM-MNC infusion in 20 people at an early stage after stroke (3–7 days). After cell transplantation, the improvement measured by modified Rankin Scale index was observed in 30% of patients after 90 days of follow-up; in the remaining 40% of the patients, an overall improvement in health was noted. There were no adverse effects of transplantation [169]. Moniche et al. conducted a phase I/II clinical study by a single-blinded study in a group of 20 patients. Five to 9 days after ischemia, 1.59 × 10^8^ autologous BM-MNCs were administered via intra-arterial route. No side effects were observed 6 months after transplantation [170]. Jiang and colleagues performed a clinical trial in four patients, three of whom underwent ischemic stroke and one had hemorrhagic stroke. Researchers transplanted 2 × 10^7^ umbilical cord-derived mesenchymal stem cells (UC-MSCs) to the middle cerebral artery. No complications were observed after 6 months. Two of three patients with brain ischemia and UC-MSCs transplantation showed an increased muscle strength and improved modified Rankin Scale index in the assessment carried out 90 and 180 days after the transplantation [171]. The advances in imaging including demonstration of safety of real-time MRI in the first clinical case may further facilitate wider adoption of intra-arterial route for the delivery of stem cells and their derivatives to the central nervous system [172].

Currently, due to the promising results obtained in preclinical studies, an application of EVs in the therapy of brain ischemia is of great interest from the clinical point of view. To date, no clinical trial of EV transplantation has been performed in patients with ischemic stroke. However, the study with transplantation of miR-124-enriched EVs isolated from mesenchymal stem cells to patients in the acute phase after cerebral ischemia is planned [158]. The growing interest in EV-based therapy among not only scientists and doctors, but also pharmaceutical companies should unfold in the near future and serve as a platform for the development of protocols for the use of EVs in clinical trials.

## Conclusions

Brain ischemia activates immune cells, which start producing pro-inflammatory cytokines and chemokines leading to blood-brain barrier disruption and further progression of tissue damage. Experimental studies revealed that mesenchymal stem cells transplantation into animal models of ischemic stroke modulate immune response; act neuroprotective; stimulate neurogenesis, oligodendrogenesis, and astrogenesis; and activate angiogenesis. Clinical trials in patients after ischemic stroke showed that MSCs do not cause significant side effects and in some trials beneficial outcome of cell transplantation was observed. Nowadays, an alternative for MSC transplantation seems to be an infusion of EVs. Experimental studies revealed that EVs have similar properties as the cells from which they are derived and they have additional advantages such as low immunogenicity or no risk of vessel blockage. The benefits resulting from EVs application will probably commence clinical trials in patients after ischemic stroke in the near future.

## Data Availability

Not applicable.

## Abbreviations

AD-MSCs: Adipose-derived mesenchymal stem cells
AMPA: α-Amino-3-hydroxy-5-methyl-4-isoxazolepropionic acid
APC: Antigen-presenting cell
ATP: Adenosine triphosphate
BBB: Blood-brain barrier
BDNF: Brain-derived neurotrophic factor
bFGF: Basic fibroblast growth factor
BM-MNCs: Bone marrow mononuclear cells
BM-MSCs: Bone marrow mesenchymal stem cells
CCL: Chemokine from the group CC (β) ligand
CNS: Central nervous system
COX-2: Cyclooxygenase
CX3CL1: Chemokine group CX3C ligand 1
CXCL2: Chemokine group CXC ligand 2
DAMPs: Damage-associated molecular patterns
DCs: Dendritic cells
DRG: Basal ganglia
EVs: Extracellular vesicles
GDNF: Glial cell-derived neurotrophic factor
GPCRs: G-protein-coupled receptor superfamily
hBM-MSCs: Human bone marrow mesenchymal stem cells
HGF: Hepatocyte growth factor
ICAM-1: Intercellular adhesion molecule 1
IFNγ: Interferon-γ
IL: Interleukin
IP-10/CXCL10: Chemokine interferon-γ inducible protein 10
MCP-1: Monocyte chemoattractant protein
MHC: Major histocompatibility complex
MIPs: Macrophage inflammatory proteins
MMPs: Matrix metalloproteinases
MRI: Magnetic resonance imaging
MSCs: Mesenchymal stem cells
MT: Mechanical thrombectomy
NeuN: Nuclear-specific protein for mature neurons
NGF: Nerve growth factor
NIHSS: National Institute of Health Stroke Scale
NK: Natural killer
NMDA: *N*-methyl-*D*-aspartate
NO: Nitric oxide
NOS: Nitric oxide synthase
NT3: Neurotrophin-3
OGD: Oxygen and glucose deprivation
RGCs: Retinal ganglion cells
ROS: Reactive oxygen species
SDF-1: Stromal cell-derived factor
STEPS: Stem Cell Therapies as an Emerging Paradigm for Stroke
SVZ: Subventricular zone
TGF-β: Transforming growth factor β
TNF-α: Tumor necrosis factor α
tPA: Tissue plasminogen activator
Tregs: Regulatory T cells
UC-MSCs: Umbilical cord-derived mesenchymal stem cells
VCAM-1: Vascular cell adhesion molecules 1
VEGF: Vascular endothelial growth factor
vWF: Von Willebrand factor
